# Development and validation of a real-time RT-PCR assay for detection of Bluetongue virus

**DOI:** 10.3389/fvets.2026.1859742

**Published:** 2026-10-21

**Authors:** Zh. K. Koshemetov, A. A. Issakhan, K. A. Shorayeva, Ye. A. Bulatov, N. K. Orazymbetova, A. K. Abubakirova, K. T. Sultankulova, M. Zh. Kaukarbayeva, Zh. B. Kondibayeva, B. K. Umuraliyev, O. N. Serikbayov, M. S. Seisenbayeva, A. K. Adalbekova, K. T. Maikhin, A. A. Abishov, K. D. Zhugunissov

**Affiliations:** 1 Research Institute for Biological Safety Problems, National Holding QazBioPharm, Gvardeyskiy, Kazakhstan; 2 National Reference Center for Veterinary, Almaty, Kazakhstan; 3 Kazakhstan Limited Liability Partnership Scientific and Production Center DiaVak-ABN, Almaty, Kazakhstan

**Keywords:** Bluetongue virus, epidemiological surveillance, field samples, molecular diagnostics, real-time RT-PCR, sensitivity, specificity

## Abstract

Bluetongue virus (BTV) poses a significant threat to livestock production, highlighting the need for highly sensitive and specific diagnostic tools for its detection. The aim of this study was to develop and validate a real-time reverse transcription polymerase chain reaction (RT-PCR) assay for the sensitive detection of BTV RNA in field samples. Four primer–probe sets targeting conserved regions of the viral genome were designed and evaluated. Among them, the BTV10S/1 primer–probe set demonstrated the highest amplification efficiency, providing stable and reproducible detection results. Reaction conditions and the composition of the PCR mixture were optimized. The analytical sensitivity of the developed assay reached 3 pg. of plasmid DNA. High specificity was confirmed by the absence of cross-reactivity with RNA from heterologous animal viruses. Clinical validation using 61 field samples collected from cattle and small ruminants showed a diagnostic sensitivity of 80%, specificity of 98.14%, and an overall diagnostic accuracy of 95.31%. The developed assay enables reliable and early detection of BTV in field conditions and can be applied in veterinary practice for epidemiological surveillance and disease control. Implementation of this method contributes to improved biosafety by facilitating timely detection and limiting the spread of infection.

## Introduction

Bluetongue (BT), caused by Bluetongue virus (BTV), is a vector-borne viral disease whose geographic distribution has significantly expanded in recent decades, affecting regions of Europe, Asia, North and South America, as well as Australia and New Zealand (1–3). In the context of globalization and climate change, the expansion of vector habitats, particularly insects of the genus Culicoides, increases the risk of virus introduction and spread into previously unaffected regions.

In the Republic of Kazakhstan, the high density of susceptible livestock populations, especially small ruminants, creates favorable conditions for rapid transmission of the infection in the event of virus introduction (2–4). Potential outbreaks of BT are associated not only with direct economic losses but also with reduced export potential of livestock products and negative impacts on the country’s epizootic status (2, 5).

Effective control of BT relies on timely diagnosis, epidemiological surveillance, and regulation of animal movement (6–8). In veterinary practice, both serological and molecular diagnostic methods are widely used. However, under endemic conditions, serological assays such as ELISA have limited diagnostic value, as they cannot reliably distinguish between active infection and post-vaccination or post-infection immunity (9–12).

Therefore, molecular diagnostic methods, particularly real-time reverse transcription polymerase chain reaction (real-time RT-PCR), are considered the most sensitive and specific tools for detecting viral RNA in clinical samples (13–17). These methods enable detection even at low viral loads and in asymptomatic cases, which is critical for early diagnosis and prevention of silent virus transmission (18–20).

Despite the availability of commercial diagnostic kits, there remains a need for the development of locally adapted molecular assays with high sensitivity, specificity, and reproducibility, taking into account the genetic variability of circulating BTV strains (19–21). This is particularly important for improving the effectiveness of epidemiological surveillance and ensuring biosafety in regions with developing livestock industries.

Accordingly, the aim of this study was to develop and validate a real-time RT-PCR assay for the sensitive and specific detection of Bluetongue virus RNA, suitable for use in veterinary diagnostic laboratories and field-based surveillance programs.

## Materials and methods

### Study design and assay description

The object of this study was a assay Real-Time RT-PCR-Blutanque-RIBSP developed at the Research Institute for Biological Safety Problems (Kazakhstan). The assay included RT-PCR reaction mixtures, primer-probe mixes, a positive control (PC), and a negative control (NC). The kit was designed to perform 50 reactions excluding control samples.

### Virus strains and specificity panel

To evaluate the analytical sensitivity and specificity of the assay, viral samples from the institute’s microorganism collection were used (Table 1). The panel included Bluetongue virus (BTV) as well as heterologous animal viruses from different families (Rhabdoviridae, Paramyxoviridae, Herpesviridae, Retroviridae, and Coronaviridae) to assess potential cross-reactivity.

**Table 1:** Viral panel used for analytical specificity evaluation of the real-time RT-PCR assay.

No.	Virus	Family (Genus)	Strain
1	Bluetongue virus	Reoviridae (*Orbivirus*)	RT/RIBSP-07/16
2	Rabies virus	Rhabdoviridae (*Lyssavirus*)	VRC-RZ2 (vaccine strain)
3	Peste des petits ruminant’s virus	Paramyxoviridae (*Morbillivirus*)	Nigeria 75/1 (vaccine strain)
4	Canine distemper virus	Paramyxoviridae (*Morbillivirus*)	LD (vaccine strain)
5	Aujeszky’s disease virus	Herpesviridae (*Alphaherpesvirinae*)	UB-95
6	Bovine leukemia virus	Retroviridae (*Deltaretrovirus*)	FLK-BLV
7	Infectious bronchitis virus	Coronaviridae (*Gammacoronavirus*)	H-120

### Field samples

Previously, during the 2024 monitoring studies, we analyzed blood samples from cattle (*n* = 40) and small ruminants (*n* = 21) collected in several regions of the Republic of Kazakhstan (Zhetysu, Almaty, Turkestan, Zhambyl, Pavlodar and North Kazakhstan regions) using the RT-PCR method in real time (22). These samples were used to evaluate the analytical sensitivity and efficiency of the Real-Time RT-PCR-Blutanque-RIBSP test system in this study (Table 2).

**Table 2:** Field samples used for validation of the real-time RT-PCR assay.

No.	Region	District	Rural district	Location / Farm	Coordinates	Animal type and age
1	Zhetysu	Koksu	Aynabulak	Aynabulak	44°37′14.0”N, 77°59′37.0″E	5 small ruminants (sheep and goats), 3–4 years
2	Zhetysu	Kerbulak	Shankhanay	Koyankoz, farm “Nurly Tan”	44°14′55.0″N, 78°14′52.4″E	5 cattle, 3–6 years
3	Almaty	Uygur	Taskarasu	Taskarasu	43°46′10.2″N, 79°27′00.8″E	5 cattle, 3–6 years
4	Almaty	Uygur	Chundzha	Chundzha	43°32′19.8″N, 79°28′03.7″E	5 small ruminants (sheep), 2–4 years
5	Turkestan	Sozak	Sholakkorgan	Sholakkorgan	43°45′53.6”N, 69°10′46.5″E	5 small ruminants (sheep), 4–5 years
6	Turkestan	Sozak	Sholakkorgan	Sholakkorgan	43°45′53.6”N, 69°10′46.5″E	4 cattle, 2.5–5 years
7	Turkestan	Saryagash	Kashar	Private household	41°27′49.2”N, 69°10′51.0″E	cattle (bull), 2.5 years
8	Zhambyl	Zhambyl	Karakemer	Karakemer village, private household	43°25′31.2″N, 77°37′19.0″E	small ruminant (sheep), 4 years
9	Zhambyl	Zhambyl	Karasu	Ashybulak village, private household	43°04′55.9”N, 71°12′05.1″E	5 cattle, 4–7 years
10	Zhambyl	Sarysu	Saudakent	Baikadam village, private household	43°44′03.5″N, 69°55′11.6″E	3 small ruminants (sheep), 4–5 years
11	Zhambyl	Sarysu	Zhayylma	Zhayylma village, private household	43°45′32.9″N, 69°47′14.9″E	small ruminant (goat), 4 years
12	Pavlodar	Maysky	Kentubek	Kentubek, farm “Akzhar Ondiris”	51°30′32.0”N, 77°29′12.0″E	10 cattle, 2–5 years
13	North Kazakhstan	Magzhan Zhumabayev	Avangard	Roshchina, farm “Bereke SKO”	54°40′12.1”N, 70°21′38.6″E	10 cattle, 2–6 years

### Ethical considerations

All animal-related procedures were carried out in accordance with internationally accepted ethical standards, including the principles of the 3Rs (Replacement, Reduction, Refinement), as well as Directive 2010/63/EU on the protection of animals used for scientific purposes (23, 24). The study protocol was approved by the Institutional Animal Ethics Committee of the Research Institute for Biological Safety Problems (Protocol No. 3).

### Primer and probe design

Nucleotide sequences were obtained from the GenBank database (NCBI). Multiple sequence alignment was performed using ClustalW and MEGA 10 to identify conserved regions of the viral genome. The VP7 gene (segment S10), known for its high level of conservation among BTV serotypes, was selected as the target (25–27).

Primers and probes were designed using Primer Express 3.0.1, Oligo Analyzer, and Primer-BLAST, with specificity confirmed using BLAST (28, 29). A TaqMan probe labeled with a 5′-FAM reporter dye and a 3′-BHQ1 quencher was used.

### Positive and negative controls

Plasmid DNA (pGEM-T-BTV, 3140 bp) containing the target fragment was used as a positive control. The plasmid was constructed using the pGEM-T Easy Vector system (Promega) according to the manufacturer’s protocol. Nuclease-free water was used as a negative control to detect contamination and prevent false-positive results.

### RNA extraction

Viral RNA was extracted using the QIAamp Viral RNA Mini Kit (Qiagen, Germany) under BSL-2 laboratory conditions (30). RNA concentration and purity were measured using a NanoDrop 2000 spectrophotometer (Thermo Scientific, USA) (31). RNA samples were stored at −20 °C until analysis.

### Real-time RT-PCR conditions

Amplification was performed using a Rotor-Gene Q real-time PCR system (Qiagen). The reaction mixture contained MMLV reverse transcriptase (8.5 U/μL), Taq DNA polymerase (0.4 U/μL), dNTPs (0.8 mM), MgSO₄ (10 mM), and specific primers and probe.

Thermal cycling conditions were as follows: reverse transcription at 50 °C for 30 min, initial denaturation at 95 °C for 15 min, followed by 45 cycles of 95 °C for 15 s and 55 °C for 30 s. Fluorescence detection (FAM channel) was performed during the annealing/extension step.

Samples were considered positive when the cycle threshold (Ct) value was <38. Samples with Ct ≥ 38 or no amplification were considered negative.

### Analytical sensitivity

Analytical sensitivity was assessed using a 10-fold serial dilution of plasmid DNA containing a 121 bp fragment of the BTV genome. The limit of detection (LOD) was defined as the lowest concentration consistently detected in replicate assays.

### Analytical specificity

Specificity was evaluated using a panel of heterologous viruses (Table 1). The absence of amplification (Ct ≥ 38 or no exponential fluorescence signal) in non-target samples indicated high assay specificity.

### Diagnostic performance evaluation

Diagnostic performance was assessed using 61 field samples and compared with a commercial reference assay (PCR-Bluetongue-Factor – prototype, Russia).

The following parameters were calculated: sensitivity (SN) = TP / (TP + FN), specificity (SP) = TN / (TN + FP), positive predictive value (PPV) = TP / (TP + FP), negative predictive value (NPV) = TN / (TN + FN), diagnostic accuracy (DA) = (TP + TN) / (TP + TN + FP + FN). Ninety-five percent confidence intervals (95% CI) were calculated using the Wilson method (32).

### Statistical analysis

Statistical analysis was performed using GraphPad Prism 10.0 (GraphPad Software, USA). Data were expressed as mean ± standard deviation (SD). Diagnostic performance metrics were calculated with 95% confidence intervals using the Clopper-Pearson method (33).

## Results

As a result of nucleotide design, four primer pairs with corresponding fluorescent probes targeting conserved regions of the Bluetongue virus genome were designed and synthesized (Table 3).

**Table 3:** Oligonucleotide primers and probes used for detection of Bluetongue virus.

No.	Primer/Probe	Sequence (5′–3′)	GC content (%)	Product size (bp)
1	BTV10/1-F	YGCRATTCACGGYGTAAA	47.2	112
	BTV10/1-R	CCGTGCAAAGTGGRCTACACR	52.4	
	BTV10/1-Probe	ATGCCAGGSCCGCTCACACG	65.0	
2	BTV10S/2-F	SCCGCTCACACGTGCKATY	57.9	119
	BTV10S/2-R	ACCGACACACCRCHYRTGTAAC	56.0	
	BTV10S/2-Probe	DCGCCGCYTAYGYRTAGYCCACT	60.1	
3	BTV-VP7-F	ACRCGWGCDATYGCDCGCG	66.7	121
	BTV-VP7-R	GTAAGTGTAATYTHAGAGACGT	35.6	
	BTV-VP7-Probe	CRCTTTGCACGGGTGTGGGTT	59.5	
4	BTV-VP7/1-F	GTTAAAAATCTHDAGAGAT	24.6	109
	BTV-VP7/1-R	GCRCATGCYCGCATYACRG	63.2	
	BTV-VP7/1-Probe	ACACTATCGCNGCAAGRGCRC	59.5	

The designed primer–probe sets (Table 3) were suitable for the detection of Bluetongue virus RNA using real-time RT-PCR.

The performance of the designed primers was evaluated using conventional RT-PCR and real-time RT-PCR. The amplification results (Figure 1) illustrate the accumulation dynamics of amplicons and allow assessment of reaction parameters, including the cycle threshold (Ct), which correlates with the initial viral RNA concentration in the sample.

**Figure 1:**
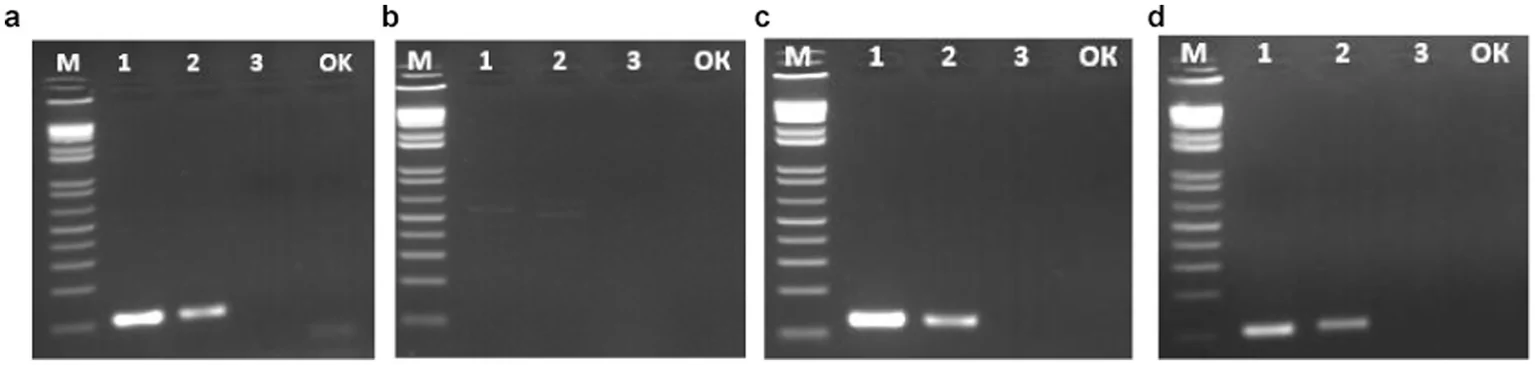
Amplification results obtained using different primer sets for detection of Bluetongue virus. **(a)** BTV10/1; **(b)** BTV10S/2; **(c)** BTV-VP7; **(d)** BTV-VP7/1. M, molecular marker (1 kb Plus DNA Ladder); 1, BTV RNA (PS cells); 2, BTV RNA (Vero cells); 3, PPR virus RNA (specificity control); NC, negative control.

Analysis of the results presented in Figure 1 showed that conventional RT-PCR using primer sets BTV10/1 (a), BTV-VP7 (c), and BTV-VP7/1 (d) produced specific amplicons of the expected VP7 gene size, whereas the BTV10S/2 primers (b) demonstrated low amplification efficiency.

The highest intensity of PCR products on agarose gel was observed for BTV10/1 and BTV-VP7 primers, indicating superior amplification performance. All tested primer sets exhibited high specificity and showed no cross-reactivity with PPR virus RNA (Figure 1).

Additional quantitative and qualitative parameters of amplification are presented in Table 4, including cycle threshold (Ct) values, amplification efficiency, and results of comparative analysis with control samples (Figure 2).

**Table 4:** Comparative amplification efficiency of different primer–probe sets.

No.	Primer–probe set	PC1 (Ct, Mean ± SD)	PC2 (Ct, Mean ± SD)	NC
1	BTV10/1	17.44 ± 0.32	19.70 ± 0.28	ND
2	BTV10S/2	40.56 ± 0.45	39.98 ± 0.40	ND
3	BTV-VP7	26.94 ± 0.41	27.70 ± 0.35	ND
4	BTV-VP7/1	ND	ND	ND

Quantitative amplification data obtained using the Cycling A. Green channel. PC1, plasmid DNA; PC2, BTV RNA (Vero cells); NC, negative control; ND, not detected.

**Figure 2:**
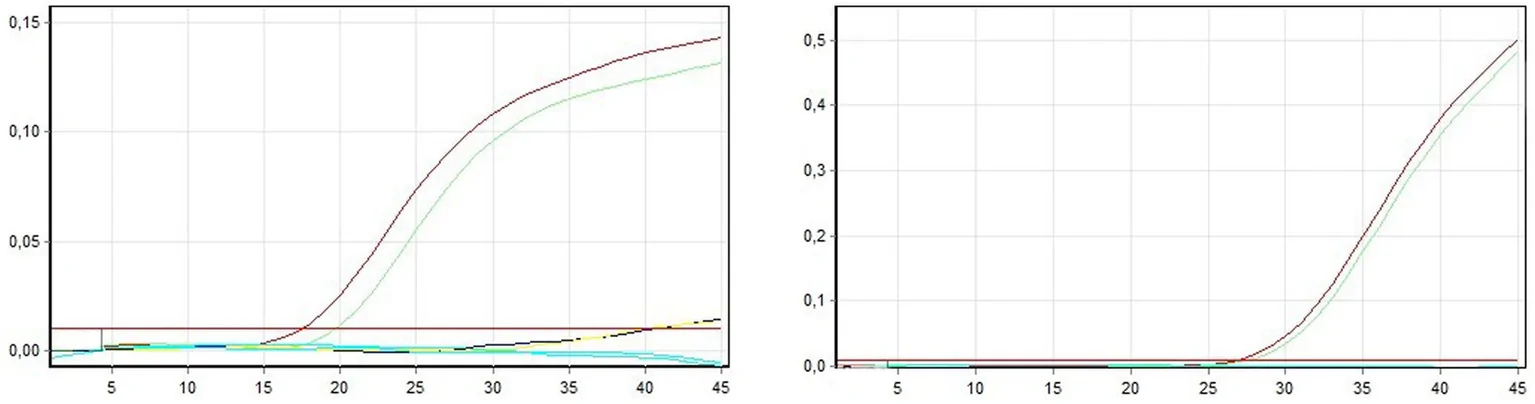
Amplification activity of different primer–probe sets for detection of Bluetongue virus.

Analysis of the results (Figure 3; Table 4) showed that the BTV10S/2 and BTV-VP7/1 primer sets did not provide efficient amplification of the target product. For BTV10S/2, high Ct values (≈40) close to the detection limit were observed, whereas no amplification was detected for BTV-VP7/1, indicating low sensitivity and limited diagnostic applicability of these primer sets.

**Figure 3:**
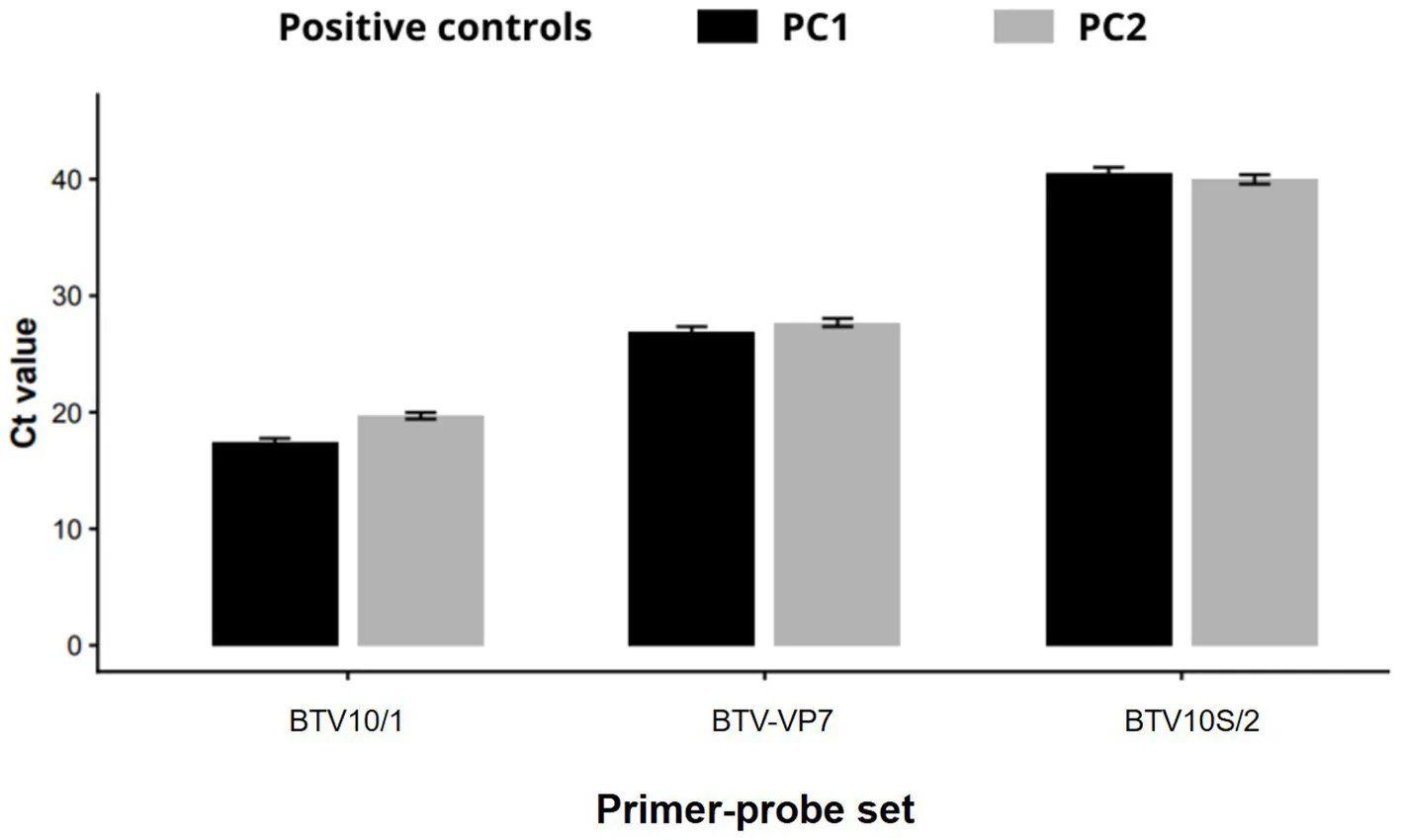
Comparative amplification efficiency of different primer–probe sets for detection of Bluetongue virus based on Ct values (mean ± SD).

Analysis of the data (Figure 3) showed that lower Ct values correspond to higher amplification efficiency. The BTV10/1 and BTV-VP7 primer sets demonstrated stable and reproducible amplification of viral RNA, whereas no amplification was observed for the BTV-VP7/1 set. Consequently, this primer set was excluded from further assay development.

Based on these results, the BTV10/1 primer set was selected as the primary diagnostic candidate due to its lower Ct values and high reproducibility across different viral RNA samples (Table 5).

**Table 5:** Characteristics of the selected BTV10/1 primer-probe set for detection of Bluetongue virus by Real-Time RT-PCR-Blutanque-RIBSP.

Target gene	Type	Tm (°C)	Length (nt)	GC content (%)	Sequence (5′–3′)	Product size (bp)
VP7	Forward	60.3	18	47.2	YGCRATTCACGGYGTAAA	112
VP7	Reverse	59.8	21	52.4	CCGTGCAAAGTGGRCTACACR	
VP7	Probe	59.5	20	65.0	ATGCCAGGSCCGCTCACACG	

The BTV10/1 primer–probe set demonstrated the highest amplification efficiency, enabling accurate detection of viral genetic material. Therefore, this set was selected as the primary diagnostic candidate for further assay development.

Probe concentration optimization was performed in the range of 50–250 nM. The results showed that concentrations of 150–250 nM обеспечивают the most stable and reproducible amplification performance (Figure 3).

Optimization of the Real-Time RT-PCR-Blutanque-RIBSP mixture allowed determination of optimal reagent concentrations: 1 U Taq polymerase, 0.2 mM dNTPs, 2.5 mM Mg^2+^, 400–600 nM primers, and 150–200 nM probe (Figure 4).

**Figure 4:**
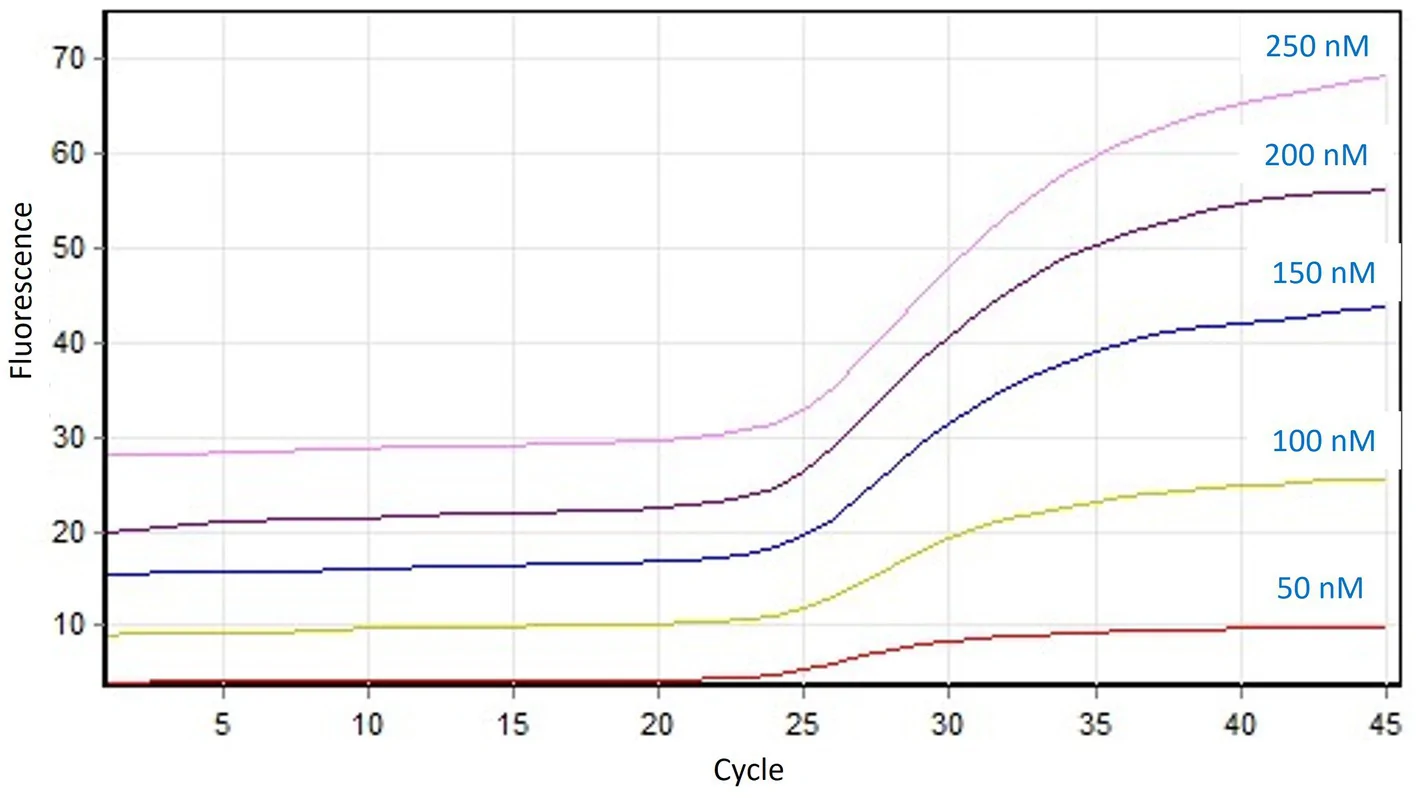
Optimization of probe concentration in real-time RT-PCR for detection of Bluetongue virus.

Analytical sensitivity was assessed using a standard sample and a series of tenfold serial dilutions. The results are presented in Table 6 and Figure 5a.

**Table 6:** Analytical sensitivity of the real-time RT-PCR-Blutanque-RIBSP assay.

No.	Dilution	Plasmid DNA concentration	Ct (replicate 1)	Ct (replicate 2)	Ct (replicate 3)	Mean Ct ± SD	CV (%)
1	10^−1^	3 μg	18.2	18.6	17.8	18.2 ± 0.40	0.45
2	10^−2^	0.3 μg	20.1	21.1	22.0	21.06 ± 0.95	2.20
3	10^−3^	3 ng	24.3	23.7	24.1	24.03 ± 0.31	1.29
4	10^−4^	0.3 ng	27.9	27.4	28.3	27.86 ± 0.45	1.29
5	10^−5^	3 pg	30.2	35.1	34.5	33.26 ± 2.66	1.61
6	10^−6^	0.3 pg	40.2	ND	ND	—	—

ND, not detected; pg, picogram; ng, nanogram; μg, microgram.

**Figure 5:**
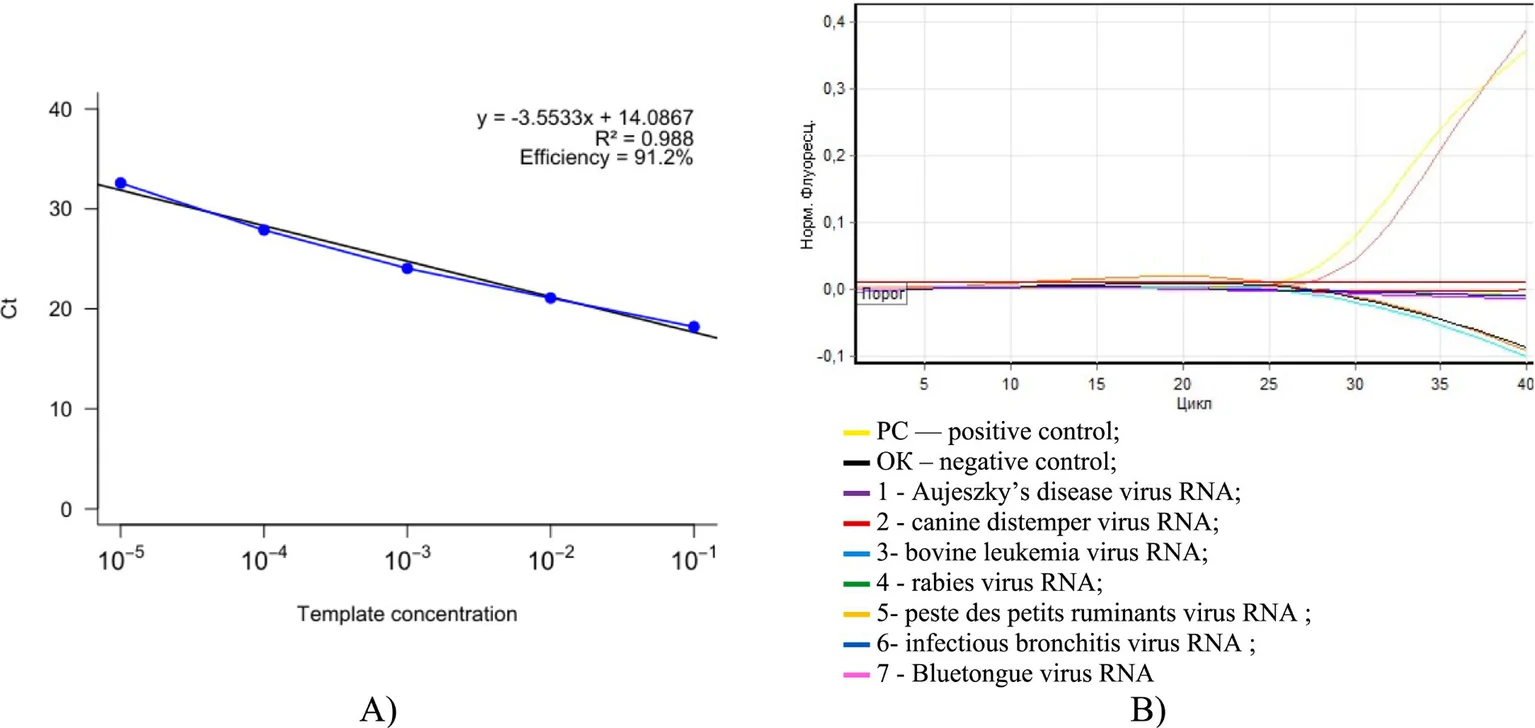
Evaluation of analytical sensitivity and specificity of the real-time RT-PCR assay. **(A)** Standard curve obtained from serial dilutions; **(B)** specificity assessment using a panel of homologous and heterologous samples.

The results demonstrated that the developed primer–probe set enables reliable detection of viral genetic material down to a concentration of 3 pg., corresponding to the limit of detection (LOD).

This level of sensitivity is critical for early diagnosis, epidemiological surveillance, and effective control of infection, allowing detection at low viral loads.

Figure 5a presents the standard curve used to evaluate the analytical sensitivity of the Real-Time RT-PCR-Blutanque-RIBSP assay. A strong linear relationship between Ct values and tenfold serial dilutions was observed, with a coefficient of determination (R^2^ = 0.988) and amplification efficiency of 91.2%.

Specificity analysis demonstrated no cross-reactivity with RNA from heterologous viruses listed in Table 1 (Materials and Methods). As shown in Figure 5b, a positive signal was detected exclusively for Bluetongue virus RNA, whereas no amplification was observed for infectious bronchitis virus, peste des petits ruminants virus, canine distemper virus, bovine leukemia virus, rabies virus, and the negative control. These results confirm the high analytical specificity of the developed assay.

Next, the samples from Table 2 (Materials and Methods) were used to evaluate the performance of the Real-Time RT-PCR-Blutanque-RIBSP method in diagnosing bluetongue (Figure 6).

**Figure 6:**
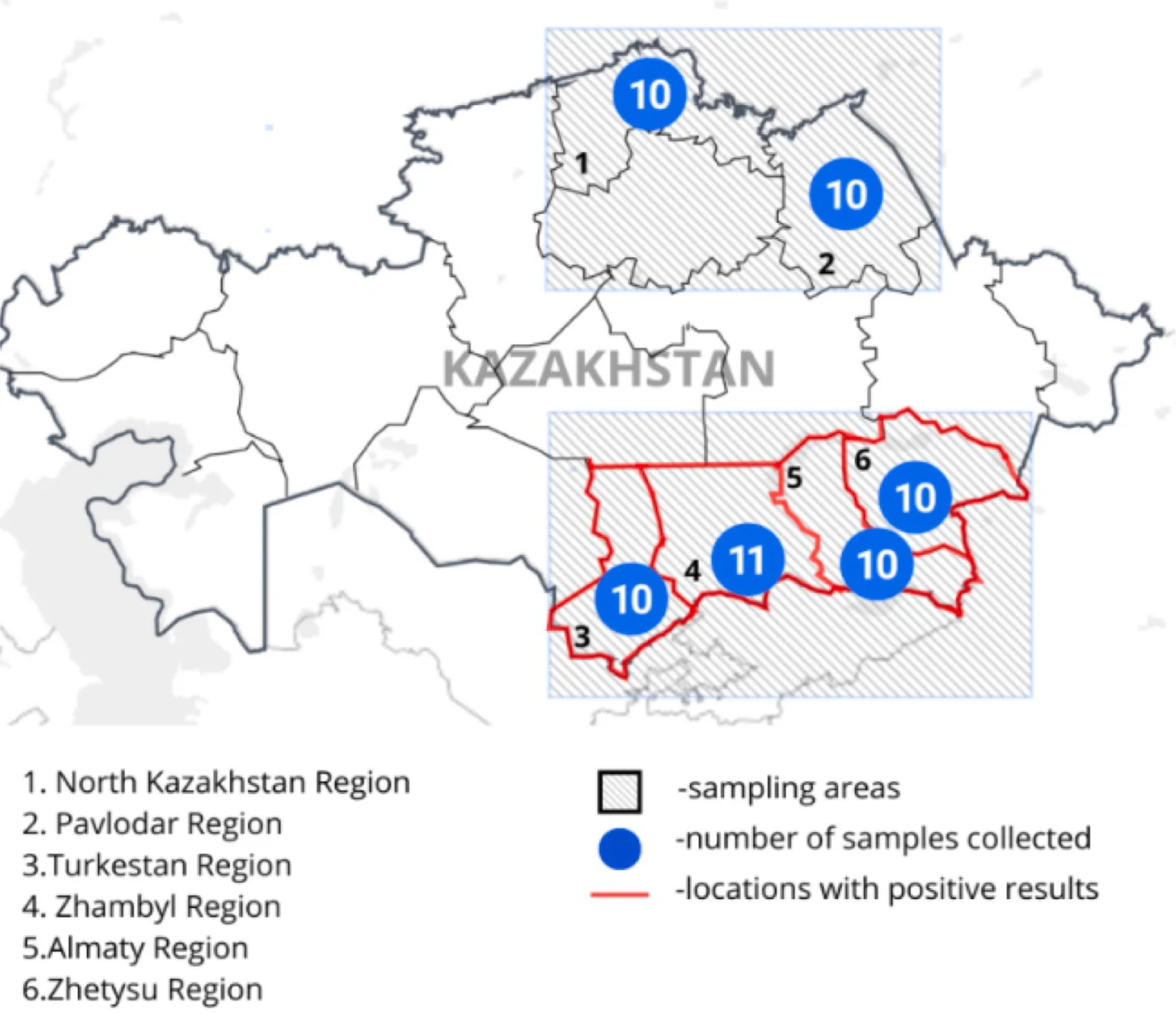
Geographic distribution of field samples analyzed by Real-Time RT-PCR-Blutanque-RIBSP across regions of the Republic of Kazakhstan. Gray markers indicate sampling locations; numbers represent the total number of analyzed samples (cattle and small ruminants) in each region; regions with positive detections are highlighted in red.

A total of 61 samples (cattle and small ruminants) were analyzed in parallel using a commercial kit (“PCR-Bluetongue-Factor”) and the developed Real-Time RT-PCR-Blutanque-RIBSP for detection of Bluetongue virus RNA (Figure 7).

**Figure 7:**
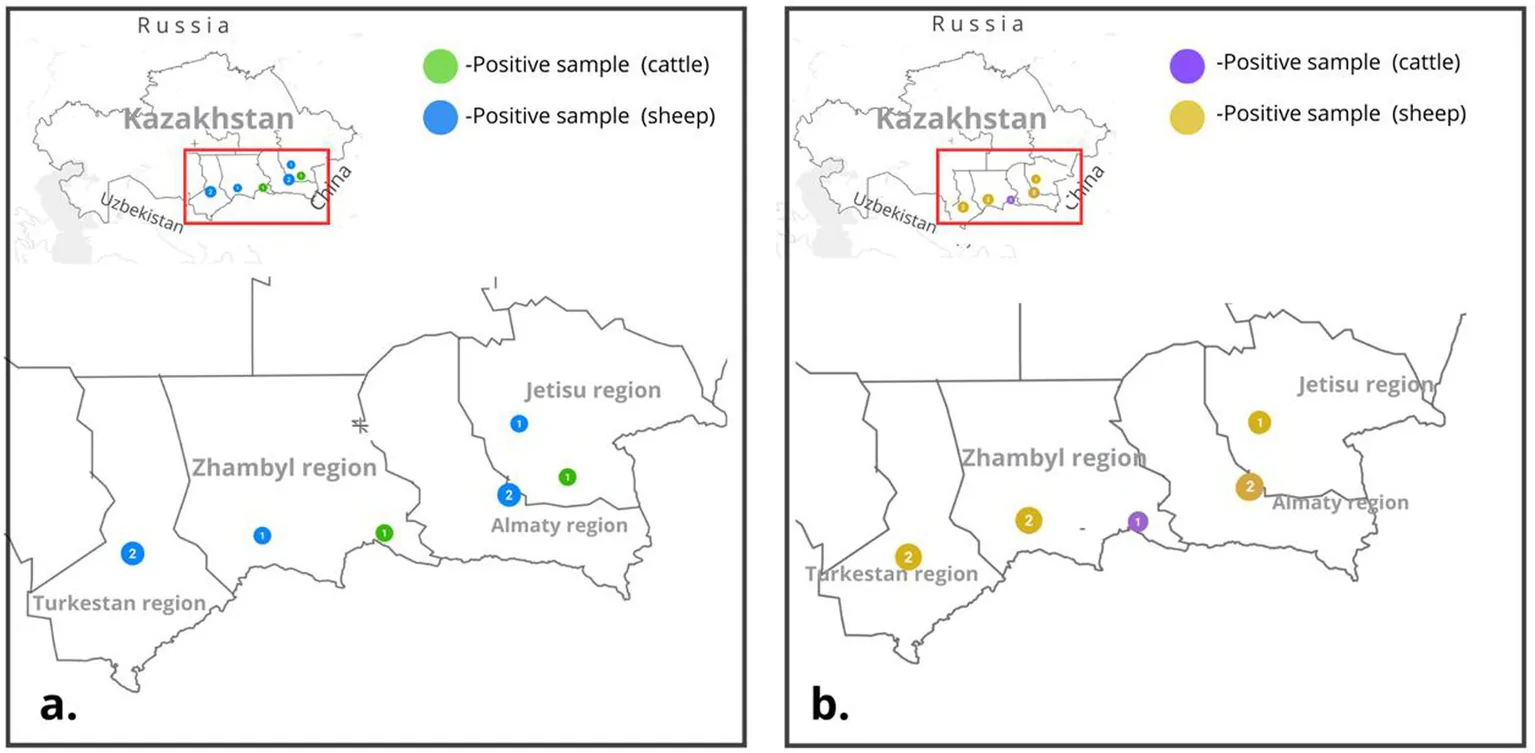
Comparative evaluation of clinical sensitivity of Real-Time RT-PCR-Blutanque-RIBSP assays for detection of Bluetongue virus and geographic distribution of positive samples across regions of Kazakhstan. **(a)** Results obtained using the commercial kit (“PCR-Bluetongue-Factor”); **(b)** results obtained using the developed assay. Marker colors indicate animal type: cattle—green **(a)** and purple **(b)**; small ruminants—blue **(a)** and yellow **(b)**. Numbers within markers represent the number of positive samples at each sampling location.

Analysis of the results (Figure 7) showed that the majority of samples were negative according to both assays. Positive detection of Bluetongue virus RNA was observed predominantly in small ruminants, whereas positive cases in cattle were sporadic.

Comparative diagnostic validation was performed using the commercial kit (“PCR-Bluetongue-Factor”) as a reference method. Overall, the results of the two assays were comparable, with only minor discrepancies. In the Almaty region (Uygur district), one cattle sample tested positive with the commercial kit but negative with the developed assay. Conversely, in the Zhambyl region (Sarysu district), one small ruminant sample tested positive with the developed assay but negative with the commercial kit.

Regional analysis indicated that positive samples were mainly detected in the Zhetysu, Turkestan, and Zhambyl regions, predominantly among small ruminants. In contrast, all samples from Pavlodar and North Kazakhstan regions were negative by both assays.

The diagnostic performance of the developed assay in comparison with the reference method is presented in Table 7.

**Table 7:** Comparative results of real-time RT-PCR assay and commercial test for detection of Bluetongue virus.

Category	Ct range	Reference assay (commercial kit)	Developed assay
Positive	10–35	8	8
False positive	35–40	1	1
False negative	>38	3	2
Negative	No Ct or >40–45	53	53
Total	—	61	61

According to Table 7, analysis of 61 samples by Real-Time RT-PCR-Blutanque-RIBSP showed that both assays yielded the same number of positive results (*n* = 8; Ct 10–35) and one false-positive result each (Ct 35–40). The number of false-negative results (Ct > 38) was 3 for the reference assay and 2 for the developed assay. The number of negative results (no Ct or Ct > 40–45) was identical for both methods (*n* = 53).

Overall, the results of the two assays were comparable, with the developed assay showing a slightly lower number of false-negative results.

Diagnostic performance was evaluated using a 2 × 2 contingency table (Table 8). Relative sensitivity, relative specificity, and overall diagnostic accuracy were calculated. Agreement between the methods was assessed using Cohen’s kappa coefficient (*κ*), and 95% confidence intervals for sensitivity and specificity were calculated using the Wilson method.

**Table 8:** 2 × 2 contingency table comparing the developed real-time RT-PCR-Blutanque-RIBSP assay with the reference method.

Developed assay	Reference (+)	Reference (−)	Total
Positive	7 (TP)	1 (FP)	8
Negative	1 (FN)	52 (TN)	53
Total	8	53	61

TP, true positive; FP, false positive; FN, false negative; TN, true negative.

Analysis using a 2 × 2 contingency table demonstrated a high level of agreement between the developed Real-Time RT-PCR-Blutanque-RIBSP assay and the reference method (Table 8). The relative sensitivity was 87.5% (7/8, 95% CI: 52.9–97.8%), and the relative specificity was 98.1% (52/53, 95% CI, 90.1–99.7%), with an overall diagnostic accuracy of 96.7% (59/61).

The Cohen’s kappa coefficient (κ = 0.86) indicated almost perfect agreement between the methods. The observed discrepancies (*n* = 2) were sporadic and likely associated with low viral loads near the detection limit of the assay.

Sensitivity, specificity, positive and negative predictive values with 95% confidence intervals, as well as overall diagnostic performance of the developed assay, are presented in Table 9.

**Table 9:** Diagnostic performance of the assays for detection of Bluetongue virus.

Parameter	Reference assay (commercial kit)	Developed assay
Sensitivity (SN, %)	72.7 (95% CI: 71.6–73.8)	80.0 (95% CI: 80–80)
Specificity (SP, %)	98.1 (95% CI: 94.8–101.4)	98.1 (95% CI: 94.8–101.4)
PPV (%)	88.9 (95% CI: 81.1–96.7)	88.9 (95% CI: 81.1–96.7)
NPV (%)	94.6 (95% CI: 89.0–100.2)	96.4 (95% CI: 91.8–101.0)

SN, sensitivity; SP, specificity; PPV, positive predictive value; NPV, negative predictive value; CI, confidence interval.

When compared with the reference method (commercial kit), the developed assay demonstrated a diagnostic sensitivity of 80% and specificity of 98.14%, whereas the reference assay showed sensitivity and specificity of 72.7 and 98.14%, respectively.

The positive predictive value (PPV) and negative predictive value (NPV) were 88.89 and 94.64% for the reference assay, and 88.89 and 96.36% for the developed assay, respectively.

The overall diagnostic accuracy of the developed assay was 95.31%.

## Discussion

Timely and accurate detection of Bluetongue virus (BTV) is a key component of veterinary epidemiological surveillance, as outbreaks of this disease are associated with significant economic losses in livestock production. Currently, molecular diagnostic methods, particularly real-time reverse transcription polymerase chain reaction (real-time RT-PCR), are considered the most sensitive and specific tools for detecting viral RNA in clinical samples (33, 34). Their application enables identification of the pathogen even at low viral loads, which is critical for early diagnosis and prevention of disease spread.

In the present study, four primer-probe sets were developed and experimentally evaluated for the detection of BTV RNA. Among them, the BTV10/1 set demonstrated the highest performance, providing stable amplification with low Ct values (17.44–19.70). These findings highlight the critical importance of primer design and validation in the development of molecular diagnostic assays. Previous studies have shown that even minor nucleotide mismatches within primer binding regions can significantly reduce amplification efficiency and lead to false-negative results (31).

Targeting conserved regions of the viral genome enabled broad detection capability of the developed assay. It is well established that genome segments 7, 9, and 10 of BTV exhibit relatively low genetic variability and are commonly used as targets in diagnostic assays (31, 33). In particular, real-time RT-PCR assays targeting segment 9 have been shown to detect multiple BTV serotypes with high analytical sensitivity (33). The results obtained in this study are consistent with these findings and support the validity of the selected target region.

The analytical sensitivity of the developed assay was determined to be 3 pg. of plasmid DNA, indicating high sensitivity. Comparable detection limits have been reported in other studies, where real-time RT-PCR assays for BTV detection typically achieve limits ranging from 10 to 100 copies of viral RNA (35, 36). Overall, real-time PCR methods provide higher sensitivity compared to conventional RT-PCR, thereby increasing the likelihood of detecting infection in samples with low viral loads (17).

The developed assay also demonstrated high analytical specificity, with no cross-reactivity observed with RNA from heterologous animal viruses. High specificity is essential to prevent false-positive results and ensure reliable laboratory diagnosis. Similar findings have been reported in other studies, where BTV-specific PCR assays showed no cross-reactivity with closely related viruses, including epizootic hemorrhagic disease virus and African horse sickness virus (17, 36).

Clinical validation demonstrated that the developed assay achieved high diagnostic performance, with a sensitivity of 80%, specificity of 98.14%, and overall diagnostic accuracy of 95.31%, which is comparable to commercially available diagnostic tests. Previous studies report that the diagnostic specificity of real-time RT-PCR assays for BTV typically exceeds 95%, confirming the reliability of this approach for routine diagnostics (17, 31).

The observed discrepancies between the developed assay and the commercial kit were minimal and likely attributable to low viral loads or genetic variability of circulating strains. It is known that at Ct values close to the detection limit, variability between assays may occur due to stochastic distribution of viral RNA molecules in the reaction mixture (35, 37).

Overall, the developed real-time RT-PCR assay demonstrated high analytical sensitivity, specificity, and diagnostic performance. The proposed method can be applied for routine laboratory diagnosis and epidemiological surveillance of Bluetongue virus. Considering the genetic variability of BTV and the potential emergence of new serotypes, periodic validation and optimization of primer sequences remain essential to ensure the reliability of molecular diagnostics (34).

This study has several limitations, including a relatively limited sample size and the absence of inter-laboratory validation. Further studies involving a larger number of samples and diverse geographical regions are required to confirm the robustness of the assay.

## Conclusion

A laboratory Real-Time RT-PCR-Blutanque-RIBSP assay for detection of Bluetongue virus RNA was developed and validated in this study. Among the four tested primer-probe sets, the BTV10S/1 set demonstrated the highest performance, обеспечивая stable amplification of the VP7 gene target with low cycle threshold (Ct 17.44–19.70) values and no signal in negative controls.

Optimized reaction conditions included 1 U Taq polymerase, 0.2 mM dNTPs, 2.5 mM Mg^2+^, 400–600 nM primers, and 150–200 nM probe. The analytical sensitivity of the assay reached 3 pg. of DNA, indicating high sensitivity. The assay also showed high analytical specificity with no cross-reactivity with heterologous viruses.

Clinical validation using 61 samples demonstrated results comparable to a commercial reference assay. The diagnostic sensitivity was 80%, specificity 98.14%, positive predictive value 88.89%, negative predictive value 96.36%, and overall diagnostic accuracy 95.31%.

In conclusion, the developed Real-Time RT-PCR-Blutanque-RIBSP assay can be effectively applied for early diagnosis and epidemiological surveillance of Bluetongue virus. Its implementation will contribute to improved biosafety through timely detection and reduced risk of disease spread. The assay may be further integrated into monitoring and control programs.

## Data Availability

The original contributions presented in the study are included in the article/supplementary material, further inquiries can be directed to the corresponding author/s.
